# Spoken Animated Self-Management Video Messages Aimed at Improving Physical Activity in People With Type 2 Diabetes: Development and Interview Study

**DOI:** 10.2196/15397

**Published:** 2020-04-23

**Authors:** Colette van het Schip, Kei Long Cheung, Stan Vluggen, Ciska Hoving, Nicolaas C Schaper, Hein de Vries

**Affiliations:** 1 Department of Physiotherapy Faculty of Health Amsterdam University of Applied Sciences Amsterdam Netherlands; 2 Department of Clinical Sciences College of Health and Life Sciences Brunel University London London United Kingdom; 3 Caphri School of Public Health and Primary Care Health Promotion Maastricht University Maastricht Netherlands; 4 Department of Endocrinology and Internal Medicine Maastricht University Medical Centre Maastricht Netherlands

**Keywords:** diabetes mellitus, type 2, internet-based intervention, telemedicine, computer tailoring, self-management, exercise, animation, health literacy

## Abstract

**Background:**

Web-based tailored interventions are a promising approach to help people with type 2 diabetes successfully adopt regular physical activity. Spoken animation seems to be effective regardless of the characteristics of the user and may be a relevant strategy to communicate complex health information

**Objective:**

The objectives of our study were to evaluate (1) pretesting communication elements and user appreciation, and (2) the applied behavior change techniques of the previously designed spoken animated video messages in a tailored self-management program for people with type 2 diabetes.

**Methods:**

We conducted semistructured interviews with patients with type 2 diabetes recruited from general practices located in different socioeconomic status urban neighborhoods. Based on the pretesting key communication elements of Salazar’s model, we asked participants about the spoken animated video messages’ attractiveness, comprehensibility, acceptance, believability, involvement, and relevance and to what extent the video messages motivated them to become more physically active. We also assessed participants’ intention to use the spoken animated video messages and to recommend them to others. To evaluate participants’ appreciation of the different applied behavior change techniques, we conducted a post hoc analysis of the qualitative data using the MAXQDA program. Transcripts were coded by 2 coders using iterative qualitative content analysis methods to uncover key health communication issues.

**Results:**

Of 23 patients who expressed an interest in participating, 17 met the inclusion criteria and 15 took part in the interviews. The positive appreciation of the comprehensibility, believability, and personalization was supported by participants’ statements on behavior change techniques and other communication elements. Reinforcement of and feedback on participants’ answers were positively evaluated as was the simplicity and concreteness of the spoken animated video messages. Most participants indicated reasons for not feeling motivated to increase their physical activity level, including being already sufficiently physically active and the presence of other impeding health factors.

**Conclusions:**

Spoken animated video messages should be simple, short, concrete, and without the use of medical terminology. Providing positive reinforcement, feedback on participants’ answers, examples that match user characteristics, and the possibility to identify with the animation figures will enhance involvement in the health message. To connect more with patients’ needs and thereby increase the perceived relevance of and motivation to use an animated video program, we suggest offering the program soon after diabetes mellitus is diagnosed. We recommend piloting behavior change techniques to identify potential resistance.

## Introduction

### Background

Type 2 diabetes mellitus is a chronic metabolic disease characterized by poor regulation of blood glucose caused by insulin resistance and beta-cell impairment [1,2]. The prevalence of people with type 2 diabetes in the Netherlands was almost 1.2 million in 2018 and is expected to increase by 34% by 2030 [3]. Socioeconomically disadvantaged and minority populations are especially at risk [4]. As a result, the cost of diabetes care in the Netherlands amounted to €1.7 billion (about US $1.9 billion) in 2011 [3]. Without proper treatment, type 2 diabetes can lead to long-term complications, such as neuropathy, nephropathy, retinopathy, cardiovascular disease, and a lowered quality of life [5]. The treatment of patients with type 2 diabetes is largely dependent on patients’ daily self-care, of which regular physical activity (PA), healthy diet, and medication adherence have been shown to be core elements [6,7]. Hence, diabetes self-management is recognized as the key factor of overall diabetes management [8]. Self-management enables patients to take control of their chronic disease by making their own decisions and performing self-chosen actions aimed at improving their health [5].

Scientific evidence shows the importance of regular PA as one way to reduce the risk of complications associated with type 2 diabetes [9,10]. Practicing regular PA may help to improve glycemic control, reduce visceral adiposity, lower plasma triglycerides, and reduce all-cause mortality risk [10]. According to the Dutch recommendations on PA, adults with type 2 diabetes should achieve at least 150 minutes of moderate- to high-intensity PA (≥3 metabolic equivalent tasks [METs]) per week spread over several days [11]. However, most people with type 2 diabetes are physically inactive and do not meet these recommendations [9]. For the Netherlands, in 2016, 40% of people with a chronic condition such as diabetes did not meet the national recommendations [12].

To promote PA self-management for people with type 2 diabetes, web-based computer-tailored support programs have been developed and can help people to improve their health behaviors and thereby their health outcomes [5,13,14]. Computer-tailored interventions are characterized by the provision of feedback (in the form of health messages) adapted to the users’ characteristics to target health-related behaviors [15]. Evidence showed that these programs can have positive effects on glycemic control, food intake, and exercise [5,13,14] and increase the effectiveness and reach of clinical-based consultations [5,16]. The relative advantage concerns the ease with which the determinants can be assessed and behavior change techniques (BCTs) can be applied in formulating web-based health messages [17]. However, many of these programs used text-driven health messages that in particular experienced problems reaching people with a low educational level, who are at risk of developing type 2 diabetes [18,19].

To improve the effectiveness of web-based self-management support programs for people with type 2 diabetes, it is a prerequisite to develop health messages that match the user experience, which thus enhances appreciation and usage of the intervention. Tailored health messages can be presented in various delivery modes, such as short message service (SMS) text messaging, video messages, or animations [20]. Exploring different delivery modes is needed, as they have the potential to enhance the effects of computer-tailored interventions. This is illustrated by several previous studies investigating the effects of video tailoring as compared with text tailoring [18,21,22]. To further explore ways to improve the user experience of these tailored interventions, more research on delivery modes is of paramount importance.

Studies showed that the delivery mode of spoken animation, consisting of simple line drawings, is a promising way to communicate complex health information to people with low health literacy [23,24]. Health literacy implies the achievement of a level of knowledge, personal skills, and confidence to take action to improve personal and community health by changing personal lifestyles and living conditions [25].

Animations may be better able than video illustrations to support the creation of an adequate mental representation to enhance learning and understanding [24,26,27]. People with low health literacy are better able to recall and have a more positive attitude toward spoken messages than to written texts [24]. Additionally, since animations do not negatively influence high–health literate target groups, health messages can be adapted to low–health literacy groups, which can lead to a better comprehension [23,24].

Several BCTs may be effective to help people with type 2 diabetes change their unhealthy lifestyles and should thus be applied in formulating health messages. A review by van Vugt et al [5] of the use of BCTs in web-based self-management programs for patients with type 2 diabetes mellitus showed that providing feedback on performance, information on consequences of behavior, barrier identification, and problem solving, and self-monitoring of behavior were linked to positive outcomes for health behavior change, psychological well-being, or clinical parameters.

According to Salazar’s model, animated health messages that are appreciated and positively evaluated on pretesting communication elements are a prerequisite to enhance the user experience of the intervention [28]. Health messages should catch the attention and be comprehensible, attractive, and relevant for the target group. BCTs target determinants of behavior and create the content of the messages. It is therefore relevant to evaluate health messages with respect to BCTs [28].

### The My Diabetes Profile Intervention

#### Development

Intervention development took place at Maastricht University. As an alternative to SMS text messaging, we previously developed tailored spoken animated health messages aimed at increasing PA behavior in people with type 2 diabetes [29]. These videos will be part of aweb-based computer-tailored program, My Diabetes Profile, supporting several parts of self-management (Dutch Trial Register NTR6840) [29].

#### Framework

**Figure 1:**
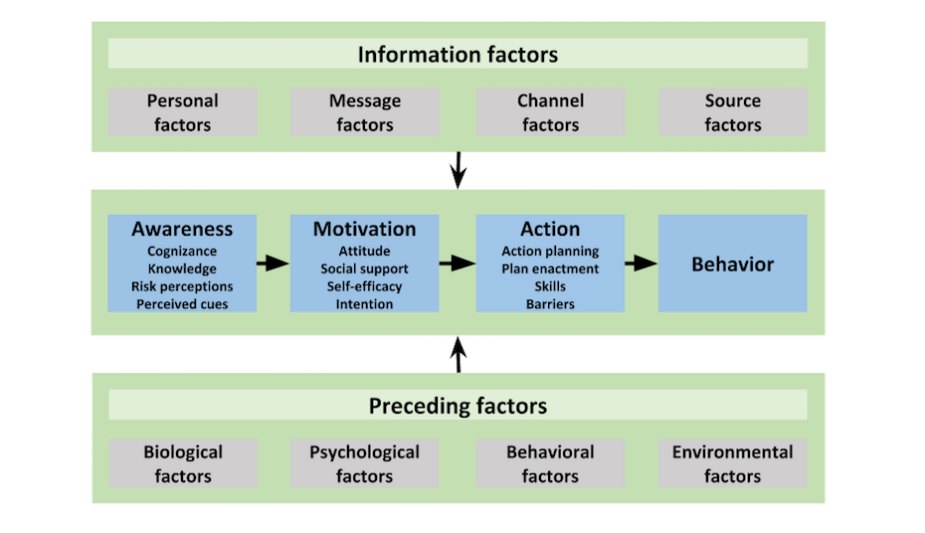
The integrated change (I-Change) model for increasing self-management behavior. Adapted from De Vries [35].

We developed tailored spoken animated video messages on the basis of the integrated change (I-Change) model [1,30,31] and tailored to address important determinants [1,32,33] of PA in patients with type 2 diabetes. We used the I-Change model [30] as the theoretical framework for developing the video messages, since it integrates several social cognitive theories, including the attitude-social influence-efficacy model [31], social cognitive theory [32], the transtheoretical model [33], the health belief model [34], and implementation and goal-setting theories (Figure 1 [35]) [32,36,37]. In recent years, the use of the I-Change model in tailored interventions has resulted in long-term positive impacts on PA behavior among adults of various age groups [1,34,36]. The model identifies relevant determinants of PA in patients with type 2 diabetes [1,22,32,33].

The I-Change model encompasses 3 phases of the behavior change process: awareness, motivation, and action. It suggests that information factors such as the quality of messages, channels, and sources that are used will determine awareness, motivation, and behavior change, which adds up to the research question. We developed the spoken animated video messages based on the 3 indicated phases in the behavior change process: (1) to increase a person’s awareness of the importance of changing behavior, (2) to motivate the person in favor of adopting regular PA, and (3) to transfer this motivation to the adoption of regular PA.

In the web-based computer-tailored program My Diabetes Profile [29], for which we developed the spoken animated video messages, users first receive a tailored health risk appraisal based on their answers to a baseline assessment (video messages on cognizance, knowledge, and risk perception). The health risk appraisal provides information on whether their perceived adherence to the PA recommendations matches with the objective guideline target. Then, if a participant does not meet the recommendations on PA, their intention to change is assessed. A participant who has a low intention to change their PA behavior is then directed to a second session that aims to increase the motivation to change (video messages on attitude, social influence, and self-efficacy) and raise awareness of the need to make improvements. If a participant has a high intention to change their PA behavior, they are directed to session 3, which shows the animated video messages on goal setting, action planning, and coping planning.

#### Construction of Spoken Animated Feedback Messages

We tailored spoken animated feedback messages according to participants’ input considering their actual level of PA and the assessment of the key determinants of PA behavior. Depending on the construct of each concept, we developed 2 (intention, preparatory plans, coping plans), 3 (knowledge, attitude, self-efficacy), 6 (cognizance, risk perception), or 9 (social influence) types of animated video messages for people with a low, a neutral, and a high score on the different determinants. For example, people could indicate on a 2-point Likert scale whether they did or did not have a coping plan (2 video messages). Attitude questions were answered on a 5-point Likert scale and recoded to a low, neutral, or high score (3 video messages). We combined cognizance, the degree to which people are aware of (the level of) their own health behavior (eg, “I am currently sufficiently physically active (yes/no)”), with a person’s actual PA behavior (meeting the norm, or almost or not meeting the norm). We selected effective BCTs based on the I-Change model and earlier tailored studies using this model [30,31] and applied them in formulating animated video messages intending to positively modify the different determinants [1,32,33]. These techniques included repeating the given answer in the questionnaire, providing information on the consequences of a behavior, identifying barriers, and solving problems [5]. Table 1 provides an overview of applied BCTs for each concept. We used the computer program Go Animate (Vyond) [38] to construct the spoken video animations. First, we wrote a script for each determinant, then developed the animations consisting of 1 or more slides per applied BCT. We asked 1 man and 2 women to voice-over the script. We took into account important requirements [19,39] for the appreciation and effectiveness of animated video-tailored health messages. These requirements implied that animated health messages should be shorter than 5 minutes, should be clear and to the point, and should be convincing and motivating [19].

**Table 1:** Overview of applied behavior change techniques for each determinant of physical activity behavior.

Concept		Behavior change techniques
All health messages		Repetition of answer Positive reinforcement (high, medium^a^) Showing empathy or respect (low)
**Determinant**		
	Cognizance and behavior	Feedback (meets the physical activity norm?) Provision of information) Persuasive communication
	Knowledge	Feedback on performance Provision of information (chunking)
	Risk perception (susceptibility and severity)	Personalized risk Provision of information on the consequences of diabetes mellitus or inactivity Problem solving, self-efficacy–enhancing conclusion
	Attitude pros	Feedback on participants’ perception of pros Counterpersuasion (low, medium) Gain framing Affective arguments Social modeling, peer reference
	Attitude cons	Counterpersuasion (low, medium) Affective and cognitive arguments Problem solving Creation of positive image
	Social influence (support, modeling, norm)	Self-efficacy–enhancing questions to enable people to mobilize social support, find modeling examples, and think about their perceived social norm Creation of positive image
	Self-efficacy	Provision of information Barrier identification Problem solving (create a plan)
	Intention	Respect of autonomy (low) Counterpersuasion (low) Provision of information on making an action plan or setting goals
	Preparatory plans	Provision of information on the pros of making a preparatory plan Provision of information on how to make a plan (tips): If-then format (goal setting) SMART^b^ principles (goal setting) Graded activity Summarizing the most important information
	Coping plans	Provision of information on how to make a coping plan (tips): If-then format (goal setting) Mobilization of social supports Summarizing the most important information

^a^Low, medium, and high refer to the specific health message in which the behavior change technique is applied for people with a low, medium, or high score on the different determinants.

^b^SMART: specific, measurable, achievable, relevant, time-bound.

### Objectives

The aim of this study was 2-fold: (1) to evaluate pretesting communication elements and user appreciation, and (2) to identify participants’ perceptions of the applied BCTs of the previously designed spoken animated video messages in a tailored self-management program for people with type 2 diabetes.

## Methods

### Study Design

According to the UK Medical Research Council framework, pilot testing is a prerequisite for the success of an intervention [40]. Hence, to optimize the intervention, we conducted a descriptive qualitative study with a cross-sectional design [41] focused on participants’ perceptions of and experiences with the previously developed spoken animated tailored video messages. To study the target group’s appreciation of the messages and to evaluate the key elements of effective communication, we conducted a semistructured interview.

### Recruitment and Study Sample

To develop effective tailored health messages for a diverse patient population, we aimed to recruit a sample that was heterogeneous in age, sex, socioeconomic status, and educational background. Data collection took place in the Dutch province of Utrecht. We selected 3 general practices located in different socioeconomic status neighborhoods in a city in the center of the Netherlands for participation. One general practitioner and 2 practice diabetes nurses agreed to recruit patients with type 2 diabetes mellitus who met the inclusion criteria. To be included, patients had to be between 40 and 70 years of age, as type 2 diabetes is relatively rare before the age of 40 years; have received a diagnosis of type 2 diabetes more than 12 months previously; and were using at least one diabetes tablet or insulin, or both. Participants also had to be able to understand and read the Dutch language and have access to a computer with internet connectivity.

Recruitment took place either during patient consultations with the practice nurse or general practitioner or by telephone. The recruiting health professionals were instructed to give a short explanation of the study and hand out the invitation letter and informed consent form to potential study participants.

### Procedure

We collected data from January to April 2017. Semistructured face-to-face interviews were conducted by 1 researcher (CvhS). To minimize bias due to the interview setting, we collected data in the participant’s natural home setting.

First, we gathered input to provide tailored advice, in line with the tailoring logic of the final intervention. One day before the interview, we sent participants a quantitative questionnaire assessing their current level of PA, as well as premotivational and motivational determinants of PA behavior derived from the I-Change model [18,37]. Questionnaires were sent by email 1 day prior to the appointment with the researcher, since we wanted to increase the chance that participants would remember the questions and their answers. We asked participants to fill in the questionnaire and send it back to the researcher that same day. Depending on the sum score (high, [medium], or low) of each concept, the corresponding spoken animated video messages were selected.

Second, a session was scheduled to first assess the demographic characteristics age, sex, marital status, nationality, educational level, and home internet use (hours per week). Subsequently, participants watched the spoken animated video messages one by one so that the researcher could ask and verify whether participants could summarize the essence of each message and thus comprehended the content. Keywords and sentences of these messages were written down by the researcher in advance and compared with participants’ answers.

Third, in the same session a semistructured interview was conducted to be able to evaluate key elements of effective communication and end users’ appreciation of the spoken animated video messages. Except for the comprehension element, we asked participants’ opinions on the pretesting elements regarding the content and design of all video messages. At the start of each interview, participants were reminded of the topic, purpose, content, added value, and procedure of the research and why it was important for them to participate. The interviews were recorded and transcribed by 1 of the authors (CvhS) to be able to analyze the content. Interviews were conducted until no new answers were being given and no new codes could be created, indicating data saturation was achieved.

### Measurement

Prior to conducting the interviews, we measured participants’ current level of PA in minutes of weekly light and moderate to vigorous PA using the Short Questionnaire to Assess Health-Enhancing Physical Activity [42]. We calculated MET scores of participants’ PA behavior based on age, PA expressed in MET values, and time spent on this PA to evaluate whether the participant met the norm on PA at the time of this study of at least half an hour of moderate- to vigorous- (≥3 or 4 METs depending on age) intensity PA on a minimum of 5 days of the week. We assessed the determinants cognizance, knowledge, risk perception (susceptibility, severity), attitude pros and cons, social influence (support, norm, modeling), self-efficacy, intention, preparatory plans, and coping plans using 3 or 4 statements per construct (eg, “My friends stimulate me to become more physically active”). We used a 5-point Likert scale (score range 1-5) to indicate the strength of agreement or disagreement, where a score of 1 corresponded to “strongly disagree” and a score of 5 corresponded to “strongly agree.” We used 10 correct or incorrect answering options to measure participants’ knowledge of type 2 diabetes mellitus. We recorded outcomes for each concept. For example, concerning attitude pro, the answer options “agree” and “strongly agree” were recoded as 1, and “strongly disagree,” “disagree,” and “neutral” scored 0. We calculated a sum score so as to assign each concept to a low, (medium), or high group. We labeled a sum score of 3 to 6 attitude pros as attitude pro high, 1 to 2 attitude pros to attitude pro medium, and 0 attitude pros to attitude pro low.

We used Salazar’s pretesting elements [28] as a frame in developing the interview guide (Table 2). Salazar argued that, while pretesting communication material, one should gather information concerning various elements; that is, a health message should catch attention, be comprehensible, be credible, be attractive, and be relevant to and accepted by the target group. Also, users should be able to identify with the content of the health communication material in order to feel personally addressed and involved. The effect of the messages on behavior (motivation) should be explored and points for improvement should be solicited to be able to enhance the content. For each pretesting element, we formulated open-ended questions on the basis of Salazar’s model [28] (eg, “What do you think of the believability of the spoken animated video messages?”). In addition, for each element we asked participants to indicate to what extent he or she perceived the animated video messages to be, for example, believable, on a score from 1 and 10, where 1 indicated “not believable at all” and 10 indicated “very believable.”

**Table 2:** Salazar’s pretesting elements [28].

Pretesting element	Recommendation	Example questions
Attractiveness	Allow participants to compare alternative versions of materials.	What do you think about the animated video messages? What was the first thing that caught your attention?
Comprehension	Try to focus participants on the main idea of the message.	Can you indicate what you think is the most important message of this animated video message(s)? What words or sentences are difficult to read or understand?
Believability	Question whether the material is credible and realistic to the audience.	What do you think about the believability of the animated video messages?
Involvement	Question whether the audience can identify with the material.	To what extent were the animated video messages tailored to your personal situation?
Acceptance	Explore issues that could potentially be overlooked.	Is there anything about the animated video messages that you find offensive or annoying?
Relevance	Have participants confirm whether the material is appropriate for them.	What type of people should read or watch this? In what way are the people in the animated video messages like or different from you?
Motivation and persuasion	Explore the effect on behavior and desires.	What does these animated video messages make you want to do? How likely are you to do that?
Improvement	Find out other ways to enhance the material.	What new information did you learn? What do you think is missing?

### Data Analysis

We computed descriptive statistics (median, mean, and range) for program rating, rating for the different key communication elements, and participants’ demographic characteristics.

We used thematic analysis to interpret the data derived from the semistructured interviews. Deductive and inductive approaches were combined and used to produce the themes and analyze the data [43]. Deductive approaches are theory driven. Analysis is shaped by preexisting theory or concepts, whereas in an inductive approach the themes identified are strongly linked to the data. This means that data are coded without trying to fit the data into a preexisting theory or framework [44].

Analysis of the data started after the first interview was conducted*.* After subsequent interviews, the transcripts were read and reread to fill in gaps or find new themes. We analyzed the content using the constant comparative method. Data derived from the semistructured interviews were managed and analyzed using MAXQDA Standard (VERBI GmbH), a software package for qualitative data analysis. We followed the stages described in the guidelines for thematic analysis developed by Clarke and Braun [44]. First, the audiotaped material was transcribed, followed by organizing, indexing, and anonymizing the data. Next, gathered data were structured using Salazar’s key elements of effective communication [28] as initial codes supplemented by other interesting features of the data. Subsequently, 1 researcher (CvhS) collated codes into potential themes, reviewed the codes, and generated a thematic map of the analysis (tree structure). Next, a second researcher (KLC) coded the data independently, whereafter both researchers discussed the coding scheme and analysis to aid data interpretation and formulate the findings. We calculated the initial interrater reliability, indicating the level of agreement or consensus between the 2 researchers, for each interview transcription, which ranged from 87.10% to 96.77%. A level of agreement of 80% is recommended as the minimum interrater reliability, whereby an interrater reliability between 82% and 100% is interpreted as almost perfect [45]. All discrepancies were solved by discussion until consensus was reached. The interview outcomes created an overview of key aspects of the spoken animated health messages that were appreciated by the target group as well as points for improvement.

## Results

### Participant Characteristics

We received contact information of 23 interested patients from the practice nurses. We telephoned these patients to explain the study, answer questions, and, for those who agreed to participate, make an appointment to conduct the interview. A total of 17 persons met the inclusion criteria, agreed to participate in the study, and were asked to complete a written informed consent form on the day of the appointment. We excluded 1 person who did not take antidiabetic medication, and 5 others decided not to participate. Reasons mentioned were not having enough time and personal circumstances.

We interviewed a total of 15 patients with type 2 diabetes mellitus, with interviews lasting between 35 and 50 minutes each. Table 3 provides an overview of the participants’ baseline characteristics. Most participants were senior adults; the median age was 63 years, with about half of them being female. About half of the participants were married or were living with a partner. The majority were Dutch; only 1 German woman participated. Their education levels were equally distributed. The median time spent using the internet at home was 7 hours per week.

**Table 3:** Participants’ baseline characteristics (N=15).

Characteristic		Value
Age (years), median (range)		63 (49-70)
**Sex, n (%)**		
	Male	7 (47)
	Female	8 (53)
**Marital status, n (%)**		
	Single	7 (47)
	Married or living with partner	8 (53)
**Nationality, n (%)**		
	Dutch	14 (93)
	German	1 (7)
**Education level, n (%)**		
	Primary school or basic vocational school	4 (27)
	Secondary vocational school or high school degree	6 (40)
	Higher professional degree or university degree	5 (33)
Home internet use (h/wk), median (range)		7 (1-24)

### Interview Outcomes

The semistructured interviews we conducted to evaluate pretesting communication elements and user appreciation (aim 1) showed a need for various improvements concerning didactics, message content, animation figures, and language use.

#### Comprehensibility

All participants mentioned that the use of animations to enhance the spoken words was highly supportive for understanding the messages, and most (8/15) indicated that the explanation of the content was simple and concrete without any difficult medical terminology, making it comprehensible to a wide audience (eg, “The explanation often is very good I think, concrete, no complex medical stories.” [male, age 61 years, patient ID D11])*.* Some (3/15) commented on the given examples as being recognizable and thereby helpful understanding the message. Except for the video message concerning cognizance, the majority of participants were able to identify the main message of each video construct, since the spoken words and sentences matched the keywords and sentences written down by the researcher in advance. Participants’ rating of the comprehensibility of the videos ranged from 7 to 10 on a 10-point Likert scale (median 8).

#### Attractiveness

Participants were asked what first attracted their attention when watching the spoken animated video messages. Some (4/15) praised the positive tone that was maintained even for people who clearly had a less healthy level of PA, while a few (3/15) mentioned the hidden jokes that attracted their attention, or the mirth of the animated video messages. Others indicated that the immediate mention of the core of the spoken animated video message was important to attract attention; for example, some participants (3/15) found it very confrontational to instantly hear all the possible consequences of diabetes, realizing they were at risk. Some participants (2/15) considered the tips on making an action or coping plan the most attractive, while 1 explicitly mentioned that the repetition of his answer (questionnaire) at the start of each spoken animated video message was important to capture his attention (“The personalized feedback at the start of the video ensures that you are drawn into the video within 10 seconds.” [male, age 61 years, D11]).

#### Animation Figures

Opinions on the animation figures varied greatly, with some participants (5/15) appreciating their diversity, as they reflected society, enabling identification, while others (2/15) noticed an absence of cultural diversity. Several participants (6/15) liked the mimics and movements of the figures because they clarify the meaning of the message. Others (2/15) had no affinity with the animation figures, as they found them old-fashioned, not modern enough. Some (4/15) thought that the animation figures fit the posture of people with type 2 diabetes mellitus, whereas others (2/15) considered them stigmatizing.

#### Text (Written and Spoken) and Voice-Overs

The font size, use of keywords, and time the text remained in the picture were mentioned as factors enhancing readability. The amount of information provided was considered “not too much and not too little.” One participant commented that, in general, each sentence contained 1 message, which for him made the content of the messages understandable and easy to follow. All participants found both male and female voices friendly and pleasant to listen to, the pace of speaking easy to follow, and words well articulated.

#### Background and Music

In general, participants (11/15) were unaware of the backgrounds and music used in the spoken animated video messages and indicated that they were not distracting but neutral, tranquil, or matching the animation*,* allowing participants to focus on the content of the message. Opinions differed on the colors used. Some preferred neutral, serene colors, while others thought that bright colors emphasized the importance of the message.

#### Duration and Amount of Video Messages

Participants (13/15) regarded the spoken animated video messages as short but powerful, to the point, and of “Facebook length.” The opinions concerning the amount of video messages varied. The majority (10/15) considered them all necessary to create an action plan, clear, educational, and crossing the t’s. However, some participants (4/15) commented that there were too many, perceiving many similarities between the different messages. One participant stated that the amount of messages in 1 animated video is more important than the total amount of animated video messages and concluded that there should be 1 clear message per video.

#### Believability

The spoken animated video message contents’ similarity to the information provided by the health professional and correspondence to the participants’ own knowledge contributed to the believability (7/14). Several participants (8/14) indicated that the “clarity,” “concreteness,” and “logical explanation” of the animated video messages made it credible to them. One person noted that the factual information provided without exaggeration or deterrent effects made it believable. Participants’ rating of the believability of the animated health messages ranged from 7 to 10 (median 9) and was graded higher than the comprehensibility.

#### Acceptance

Some participants (2/15) felt that the spoken animated video message on social influence was compelling and an interference to their own life. Others (2/15) thought that the question “If you would get these complaints how disagreeable would you find that?,” which queries the perceived severity as part of risk perception, was strange and rhetorical (eg, “Of course I’ll find it disagreeable!” [female, age 59 years, D14]). A few (2/15) indicated that the spoken animated video messages contained many fat animation figures, which they considered stigmatizing. One participant felt annoyed by the repetition of the same message “over and over again.”

#### Involvement (Personalization)

Most participants (10/15) indicated that they recognized themselves in the feedback on their questionnaire responses, the examples provided, or the animation figures, whereby they felt personally addressed (eg, “The confirmation, since the videos you showed were really like ‘you filled in the questions this way’...that was confirmed in the movie...so you feel...I’m watching something that really addresses me.” [female, age 66 years, D02]). Participants’ rating for the level of personalization of the spoken animated video messages ranged from 6 to 10 (median 8).

#### Relevance (Added Value)

Most participants (9/15) indicated that, due to the absence of new information or their current level of PA, the spoken animated video messages did not meet their needs. However, other interviewees (6/15) confirmed that the spoken animated video messages fit their needs, since watching them raised their awareness of the importance of PA in relation to type 2 diabetes and made them understand they were at risk. Some (2/15) stated that the video messages provided a good insight into type 2 diabetes and, therefore, are especially relevant for people who have just had type 2 diabetes diagnosed. Some participants (3/15) confirmed that the spoken animated video messages fit their needs, pointing out the power of repeating the message. Participants’ rating of the relevance (added value) of the animated video messages to their health ranged from 6 to 10 (median 8).

#### Motivation and Persuasion

Some participants indicated that the spoken animated video messages did not motivate them to increase their level of PA, since they were either already sufficiently physically active, meeting Dutch PA recommendations (5/15), or were impeded by other health factors such as low back pain, “weak knees,” or fibromyalgia, even though they were convinced of its importance (3/15). Those who did feel motivated by the video messages to change their PA behavior mentioned the severity of the possible consequences of type 2 diabetes (2/15) and the importance of PA for their health (3/15) as key reasons. Participants’ rating of the extent to which the spoken animated video messages motivated them to become more physically active ranged from 1 to 10 (median 5).

### Points for Improvement

We divided suggestions for improvement into 5 main topics: animation figures used, didactics, message content in general, specific video messages, and language use. Animation figures were seen as old-fashioned (2/15), too much unilaterally emphasizing the relationship between overweight and diabetes (2/15), or containing too little cultural diversity (2/15). Suggested didactic improvements were providing an overview of upcoming animated video topics at the start of the video program (1/15) and ending with a summary of the content of each animated video message (2/15). Others suggested adding examples for people who are single or do not have children (2/15) and using testimonials (1/15). Two participants suggested that by standardizing the amount of PA, people could see 30 minutes per day as a maximum instead of a minimum. One suggested adding more concrete personalized outcome measures, whereas another suggested linking the creation of an action plan to a direct concrete result. One participants suggested avoiding the use of real names in the examples and replacing them with “friend” or “partner” (1/15). One participant suggested explaining and emphasizing the link with nutrition more in the animated video messages on PA, and explaining PA more in the video messages on nutrition, despite the fact that there is a separate module on nutrition. Suggestions for improving the content of specific animated video messages concerned the number of messages given simultaneously in 1 video and the amount of information on 1 screen. Two participants perceived the question “If you would get these complaints how disagreeable would you find that?” as strange and rhetorical. Two others occasionally experienced the language use as pushy (“It is a bit like ‘you must do this, you will do that,’ a little compulsive.”).

### Intention to Use the Spoken Animated Video Messages as a Tool to Increase Physical Activity Behavior

Several participants indicated they did not intend to watch the spoken animated video messages again, since they either were already sufficiently physically active (5/15), had sufficient knowledge concerning diabetes (4/15), or preferred the assistance of a medical professional (1/15). Of those participants who expressed the intention to use the video messages as a tool, some (6/15) thought they would use the video program as a manual or guideline (eg, “...the guidelines that it provides to handle it proactively, and an individual concretization.” [male, age 66 years, D05]). Participants’ rating of their intention to use the spoken animated video messages as a tool to change their PA behavior ranged from 1 to 10 (median 6).

### Intention to Recommend the Spoken Animated Video Messages to Others

Close to half (6/15) would recommend the video messages to important others and thought that the messages would especially be helpful for people with a new diagnosis of type 2 diabetes, since it would provide them with important information at an early stage. Due to time pressure during a consultation, there is not always time to explain things well, they commented. Some interviewees (4/15) mentioned ignorance or unawareness of the possible consequences of type 2 diabetes, among others, as reasons to recommend the spoken animated video messages, while others (5/15) intended to recommend the video messages to motivate people to change their lifestyle, and 1 participant mentioned the concreteness and individual approach as reasons for recommending them to others. Participants’ rating of their intention to recommend the spoken animated video messages to important others ranged from 5 to 10 (median 8).

### Evaluation of the Behavior Change Techniques

To evaluate participants’ appreciation of the different applied BCTs (aim 2), we conducted a post hoc analysis of the qualitative data.

Participants (10/15) thought that the provided personalized feedback on their answers or performance immediately made the message personal and drew their attention. All participants appreciated the positive reinforcement, mentioning the friendliness of speech, and some (5/15) mentioned the positive tone throughout. Most participants recognized the information provided but commented that they did not hear any new information. All participants were convinced of the importance of information on the consequences of behavior and type 2 diabetes mellitus, and some (4/15) considered the information provided to be quite confrontational. The majority (13/15) acknowledged the relevance of mobilizing social support, although some participants (2/15) disliked the idea of others interfering with their lives and behavior and were not interested in what other people think or do. The gain-framing arguments used to persuade people to adopt a positive attitude toward PA behavior were confirmed by all participants, as well as the arguments used to counterpersuade participants who had a negative attitude toward PA. Not all participants agreed on the affective arguments used. One did not recognize being proud after being physically active, and others (4/15) did not feel fitter or more relaxed or disliked PA. The majority of participants (11/15) thought that the provided tips on how to make an action plan and coping plan were very clear, concrete, and helpful to become more physically active and overcome barriers. Especially the provided tips and examples on concrete goal setting, the if-then concept, the SMART (specific, measurable, achievable, relevant, time-bound) principle, and the tip on graded activity were seen as necessary for successful behavior change and maintenance. Only a few participants (2/15) said that the spoken animated video messages motivated them to become more physically active and to change their PA behavior systematically. Fewer than half of the participants stated that they did not like making an action plan (2/15) or did not need a plan, since they already embedded PA in their life and were sufficiently active (5/15). All participants recognized the provided examples of possible barriers, but varied in their perceived need to overcome these barriers by making a coping plan.

## Discussion

### Principal Findings

The aim of this study was 2-fold: (1) to evaluate pretesting communication elements and user appreciation, and (2) to evaluate the applied BCTs of the previously designed spoken animated video messages in a tailored self-management program for people with type 2 diabetes.

To evaluate pretesting communication elements and user appreciation (aim 1), we formulated interview questions. We used the pretesting elements as defined by Salazar [28] as a framework, covering attractiveness, comprehensibility, acceptance, believability, involvement, relevance, motivation, and improvement. To evaluate the applied BCTs (aim 2), we performed a post hoc analysis. We found a need for various improvements concerning didactics, message content, animation figures, and language used. The results of this study provide insights into what key communication elements are appreciated and what BCTs should be taken into account when designing spoken animated tailored video messages.

In general, the evaluation of the pretesting communication elements and user appreciation (aim 1) showed that participants appreciated the spoken animated video messages for being to the point, short but powerful, and positive in tone.

The comprehensibility, believability, involvement (personalization), and perceived relevance of the video program to participants’ health received high mean scores. Participants noted that simplicity, concreteness, and the absence of difficult medical terminology made the spoken animated video messages comprehensible. The use of animations to enhance the spoken words was perceived as highly supportive for understanding the messages, confirming literature indicating that spoken animation is a promising way to communicate complex health information [23,24]. The fact that the content of the spoken animated video messages corresponded to the participant’s knowledge of type 2 diabetes and was similar to the information provided by health professionals made the health messages credible to most participants.

The repetition of a participant’s answer, the given examples, and the possibility to identify with the animation figures made participants feel personally addressed. According to the elaboration likelihood model [46], providing tailored feedback results in more thoughtful information processing via the central route of persuasion, since tailored messages are perceived as being personally relevant and thus encourage the person to pursue the desired behavior [30]. Moreover, the use of role models, enabling identification, is a common effective method to increase attention, remembrance, self-efficacy, and skills [47,48].

Although participants were convinced that the use of the spoken animated video messages as a tool to change their PA behavior could be relevant to their health, many indicated that the animated video messages did not motivate them to become more physically active and thus did not match their needs. Reasons mentioned were the presence of other impeding health factors limiting PA participation, being sufficiently active already, or the absence of new information. In line with this, a previous study showed that the presence of comorbidities was negatively associated with PA participation in patients who had had type 2 diabetes for more than 1 year, but not in those with a new diagnosis [49]. Also, the fact some participants found the content relevant, while others did not, is in line with the I-Change model [37], which states that individuals move through stages of change.

The spoken animated video program was thought to be especially relevant for people with a new diagnosis of type 2 diabetes, since it provides them with important information at an early stage. These arguments suggest the importance of formulating inclusion criteria for participation and considering when the program is offered after diagnosis in order to connect more with a patient’s needs and abilities and thereby increase the perceived relevance, usage, and PA behavior.

Following the evaluation of the applied BCTs (aim 2), a few participants expressed negative opinions on the use of some BCTs in the messages. For example, 2 participants did not appreciate the video message on social influence, considering themselves responsible for their own behavior (change), and didn’t want anyone to interfere, indicating the importance of a well-thought-out formulation of a video message. Also, where the majority appreciated the BCT concerning action planning, and regarded the provided tips as helpful to change their behavior in a systematic way, others did not like or did not feel a need to make a plan. These examples may suggest the importance of the selection of BCTs based on their motivational level [50] and the users’ preferences [51].

### Limitations

We recruited a heterogeneous study population with well-distributed baseline characteristics of sex, marital status, and educational level. Hence, the insights obtained allowed us to develop spoken animated tailored video messages for a diverse patient population. Yet there was potentially a response bias, reflected by the small cultural diversity, as 13 participants were Dutch and 1 was German. Since Moroccans and Turkish people form a large part of our target population, the results of this pretest might not be applicable to this group.

Some of the results might be biased by the likelihood that only more interested and motivated patients participated in the study, which may affect the generalizability. Other differences in characteristics between participants and patients who did not participate, such as disease severity and other impeding health factors, could also have contributed to biased results. For instance, 3 participants indicated that, due to impeding health factors, they were not able to become more physically active, although they were motivated and wanted to. Further, a large number indicated that they had not yet experienced any serious consequences of diabetes, and were sufficiently active and therefore not motivated to increase their level of PA.

Although the use of thematic analysis to analyze the data may have limited the objectivity of this study, the use of Salazar’s pretesting elements enabled us to evaluate key elements of effective communication. Although we based most of the conclusions regarding our research question on qualitative data, the addition of quantitative measures (grades) may have yielded complementary information and valuable insight into the appreciation of the developed animated video messages and their compliance with the conditions of effective communication. Lessons learned concerning the strengths and limitations of our approach may help to improve the spoken animated video messages and thereby the electronic health self-management program and its integration into diabetes care.

### Conclusion

We conducted this study (1) to evaluate key elements of effective communication and user appreciation, and (2) to evaluate the applied BCTs of the designed spoken animated health messages in a tailored self-management program, in order to enhance the delivery of health messages, which in turn may enhance the user experience and usage of the tailored self-management program for patients with type 2 diabetes mellitus. Our evaluation using Salazar’s pretesting elements identified several key elements that enhanced appreciation of the animated tailored health messages, as well as points for improvement. The high appreciation of the pretesting elements’ comprehensibility, believability, and personalization was supported by participants’ statements on BCTs and other elements that help the creation of animated tailored health messages. Based on the results of this study, we recommend that health messages be simple, concrete, to the point, short but powerful, and positive in tone, and lack difficult medical terminology. The use of animations to enhance the spoken words was perceived as highly supportive for the understanding of health messages. Providing positive reinforcement, feedback on participants’ answers, and examples that match user characteristics and the possibility to identify with the animation figures contributes to participants’ involvement in a health message. To connect more with patient’s needs and physical abilities—and thereby increase the perceived relevance and motivation to use an animated video program, and thus increase PA participation—when developing spoken animated health messages we suggest taking users’ characteristics and preferences into account, basing the selection of BCTs on the stages of change, and considering when the program is offered after diagnosis.

## Abbreviations

BCT: behavior change technique
I-Change: integrated change
MET: metabolic equivalent task
PA: physical activity
SMART: specific, measurable, achievable, relevant, time-bound
SMS: short message service
